# Trametinib with or without Vemurafenib in BRAF Mutated Non-Small Cell Lung Cancer

**DOI:** 10.1371/journal.pone.0118210

**Published:** 2015-02-23

**Authors:** Monika Joshi, Shawn J. Rice, Xin Liu, Bruce Miller, Chandra P. Belani

**Affiliations:** Penn State Hershey Cancer Institute, Hershey, Pennsylvania, United States of America; Roswell Park Cancer Institute, UNITED STATES

## Abstract

V-Raf Murine Sarcoma Viral Oncogene Homolog B (BRAF) mutated lung cancer is relatively aggressive and is resistant to currently available therapies. In a recent phase II study for patients with BRAF-V600E non-small cell lung cancer (NSCLC), BRAF V600E inhibitor demonstrated evidence of activity, but 30% of this selected group progressed while on treatment, suggesting a need for developing alternative strategies. We tested two different options to enhance the efficacy of vemurafenib (BRAF V600E inhibitor) in BRAF mutated NSCLC. The first option was the addition of erlotinib to vemurafenib to see whether the combination provided synergy. The second was to induce MEK inhibition (downstream of RAF) with trametinib (MEK inhibitor). We found that the combination of vemurafenib and erlotinib was not synergistic to the inhibition of p-ERK signaling in BRAF-V600E cells. Vemurafenib caused significant apoptosis, G1 arrest and upregulation of BIM in BRAF-V600 cells. Trametinib was effective as a single agent in BRAF mutated cells, either V600E or non-V600E. Finally, the combination of vemurafenib and trametinib caused a small but significant increase in apoptosis as well as a significant upregulation of BIM when compared to either single agent. Thus, hinting at the possibility of utilizing a combinational approach for the management of this group of patients. Importantly, trametinib alone caused upregulation of p-AKT in BRAF non-V600 mutated cells, while this effect was nullified with the combination. This finding suggests that, the combination of a MEK inhibitor with a BRAF inhibitor will be more efficacious in the clinical setting for patients with BRAF mutated NSCLC.

## Introduction

A majority of patients with non-small cell lung cancer (NSCLC) are diagnosed at a later stage and currently available treatments including chemotherapy and radiotherapy seem to be insufficient in beating this deadly disease. The presence of an activating mutation in the epidermal growth factor receptor (EGFR) is associated with high response rates and improved progression free survival (PFS) with the use of EGFR tyrosine kinase inhibitors (TKIs) [1–3]. Erlotinib and gefitinib are first generation TKIs that cause reversible inhibition of the tyrosine kinase domain of EGFR. Erlotinib was initially approved for clinical use in advanced NSCLC in the second line setting on the basis of positive results of the phase 3 BR.21 trial [4]. Phase 3 trials, like OPTIMAL (erlotinib versus chemotherapy as first-line treatment for patients with advanced EGFR mutation-positive non-small-cell lung cancer)[5] and IPASS (Iressa Pan Asia Study) [6], have shown clear improvements in response rates and progression free survival (PFS) with first generation TKIs in the first line setting when compared to traditional platinum based chemotherapy. This has already led to the use of EGFR TKIs as a first-line therapy for patients with NSCLC harboring a sensitizing EGFR mutation as opposed to standard combination chemotherapy. Similarly, BRAF (v-Raf murine sarcoma viral oncogene homolog B1) mutation can also drive tumor development in NSCLC. Mutations in the *BRAF* gene have a frequency of ∼2–3% in NSCLC [7,8]. A V600E mutation on exon 15 comprises approximately 50% of all BRAF mutations while non-V600E BRAF mutations make up the rest 50% [9].

BRAF mutant NSCLC is thought to be aggressive and show resistance to currently available therapies [10]. Vemurafenib is an oral BRAF inhibitor selective for the V600E mutation, and has been proven to be effective in treating advanced melanoma patients with the same mutation. There is evidence to support that BRAF inhibitors, used alone in NSCLC with BRAF-V600E positive mutation, demonstrate only a modest benefit with no complete responses, 40% partial response, and 30% with disease progression while on treatment [11]. The effect of MEK (MAPK/ERK Kinase) inhibition in BRAF mutant NSCLC has not been thoroughly investigated and a recent study showed that the combination of a BRAF inhibitor (dabrafenib) and MEK inhibitor (trametinib) was also effective in treating advanced melanoma [12]. Trametinib is an orally available MEK inhibitor recently approved by Food and Drug Administration (FDA) for use in treating metastatic melanoma in patients with BRAF-V600E and BRAF-V600K mutations, based upon results of a clinical trial by Flaherty *et al.*, showing a significant benefit in both progression free and overall survival [13].

In the preclinical setting, receptor tyrosine kinase (RTK) inhibition has a dominant effect on suppression of phosphoinositide 3-kinase (PI3K) signaling pathway. One of the mechanisms of resistance for RTK inhibition in KRAS mutant cell lines is through activation of the RAS signaling pathway [14]. A colon cancer cell line with a BRAF V600E mutation was shown to escape vemurafenib inhibition by activating the epithelial growth factor receptor (EGFR); however, treatment of these cells with vemurafenib and an EGFR inhibitor prevented vemurafenib resistance and induced apoptosis [15]. We hypothesize that mutation in the BRAF pathway causes hyper-activation of ERK and thus, combining EGFR inhibition with BRAF inhibition would be of benefit. We also set out to determine if the MEK inhibitor trametinib would sensitize the BRAF mutated wild type (WT) EGFR NSCLC cells to BRAF inhibition by vemurafenib. We hypothesized that BRAF-V600E cells have limited sensitivity to the BRAF inhibitor because of activation of the MAPK pathway [12]. Hence, trametinib could enhance the efficacy of a BRAF inhibitor in BRAF mutated NSCLC. Resistance to BRAF inhibitors is mediated through multiple mechanisms inducing reactivation of MAPK pathway [16–19]. Trametinib targets MEK, which is downstream of BRAF in the MAPK pathway. Hence, combining trametinib with a BRAF inhibitor, either vemurafenib or dabrafenib, would be more effective. Our aim was to investigate if trametinib and vemurafenib could cooperate to suppress the ERK/MAPK signaling pathway in BRAF mutant NSCLC cell lines. We compared the efficacy of single agents, vemurafenib or trametinib, to that of the combination of vemurafenib plus trametinib in BRAF mutated NSCLC cell lines, HCC364 [V600E-BRAF, WT EGFR] and H1755 [non-V600E, G469A BRAF mutant, WT EGFR].

## Materials and Methods

### Cell lines, reagents and antibodies

A549, H460, H1755 were obtained from American Type Culture Collection (ATCC, Manasses, VA). Whereas, the HCC364 cell line was obtained from Dr. Adi F. Gazdar’s research laboratory, University of Texas, Southwestern Medical Center (Dallas, TX) [20]. All cells were cultured in RPMI 1640 medium supplemented with 10% fetal bovine serum, streptomycin, and penicillin at 37°C with 5% CO_2_. BRAF V600E and G469A mutations in HCC364 and H1755 cells, respectively, were confirmed using a SNaPshot fragment analysis method according to the protocol developed by Su et al. (S1 Fig.) [21]. Antibodies were purchased from Cell Signaling Technologies (Danvers, MA). Erlotinib, vemurafenib and trametinib were obtained from Selleckchem (Houston, TX).

### Cell proliferation and long-term growth assays

Cells were seeded at 1x10^4^ cells per well in a 96-well tissue culture plate. The following day, cells were treated with erlotinib, trametinib, vemurafenib, or combinations as indicated in figure legends. Dimethyl Sulfoxide (DMSO) was used as a vehicle control. Cell proliferation was measured 48 or 72 hours post drug treatment using a Cell Titer 96 aqueous nonradioactive cell proliferation assay (Promega, Madison WI) according to the manufacturers’ protocol. Relative viability was calculated for each well by subtracting background absorbance prior to normalization to vehicle control treated wells.

Long-term growth assays were performed by seeding cells at 1500 cells per well on a 12 well plate. Drugs were added directly to each well the following day, and fresh media with drug was added after 4 days. After 7 days, cells were washed with PBS, fixed in 2% paraformaldehyde for 20 minutes at room temperature (RT), incubated with 70% ethanol for 5 minutes, and then stained with 0.1% trypan blue for 60 minutes at RT. After destaining in PBS three times, wells were scanned. Afterwards, the dye was solubilized in 1% SDS and the absorbance was measured at 590 nm for three 100 μl aliquots from each well on a Synergy HT plate reader (BioTek, Shoreline WA). Background absorbance from a well without cells was subtracted from experimental values, and experimental values were normalized to vehicle treatment.

### Immunoblot assay

Proteins were harvested from cells in a lysis buffer containing 40mM Tris.Cl pH7.6, 1% Triton X-100, 1% deoxycholate, 150 mM NaCl plus protease and phosphatase inhibitor cocktails (Sigma-Aldrich, St. Louis, MO) at 4°C for 30min. Total protein was quantified with a BCA kit (Thermo Scientific, Waltham MA), and equal amounts of protein were partitioned through 10% SDS-PAGE gels, electroblotted to PVDF, and blocked with 5% NFDM. Blocked membranes were incubated overnight with primary antibodies, with appropriate HRP-conjugated secondary antibody, prior to chemiluminescent detection.

### Flow cytometry

Apoptosis was assayed by staining cells with FITC-labeled Annexin-V (BD Pharmingen, San Jose CA)) and ViaProbe (7-AAD, BD Pharmagen) prior to analysis on a Calibur flow cytometer (BD Biosciences, San Jose CA). Cells were seeded in triplicate onto a 6-well plate, and the following days cells were treated with the indicated drugs. Paclitaxel was used as a positive control for apoptosis experiments. Twenty-four to 48 hours after treatments, floating cells and adherent cells were harvested then stained with Annexin-V-FITC and 7-AAD in 1x annexin V-binding buffer (BD Biosciences) for 30 minutes at room temperature in the dark. Fluorescence was measured with a flow cytometer, and resulting data was analyzed with WinMDI 2.8 software (The Scripps Institute, http://facs.scripps.edu/software.html).

Cell cycle analysis was performed by ethidium bromide staining. Cells were seeded in triplicate onto a 6-well plate a day before drug treatment. After 24 hours, nuclei were harvested and stained in a hypotonic lysis solution (7 mM sodium citrate, 0.2% Triton X-100) with ethidium bromide (50 ng/ml) and RNase A (50 μg/ml) for 30 minutes in the dark. Fluorescence was measured on a Calibur Flow Cytometer. The acquired data were analyzed using ModFitLT 3.2 software (Verity Software House, Topsham ME).

### Statistical analysis

The statistical significance of differences between two groups or among multiple groups was analyzed with two-sided unpaired Student's t-tests (for equal variances) as implemented by Excel 2007 (Microsoft Corp., Redmond WA). All data shown are mean SEM of triplicate values from three separate experiments. *p < 0.05, **p < 0.01, and ***p <0.001 as compared with the control group. Independent Student’s t-tests or one-way ANOVA were used to compare the continuous variables between the two groups or more than two groups. Results were considered to be statistically significant at p<0.05. The cytotoxic synergy was analyzed and graphed with the use of CalcuSyn software (BioSoft, Cambridge UK) and was expressed as the combination index at the LC99 (i.e., concentration lethal to 99% of cells). Further confirmation was obtained with in a 5x5 matrix using a CellTiter-Glo assay and Bliss additive model analysis.

## Results

### Expression of p-ERK and p-AKT signals in NSCLC cells

**Fig 1:**
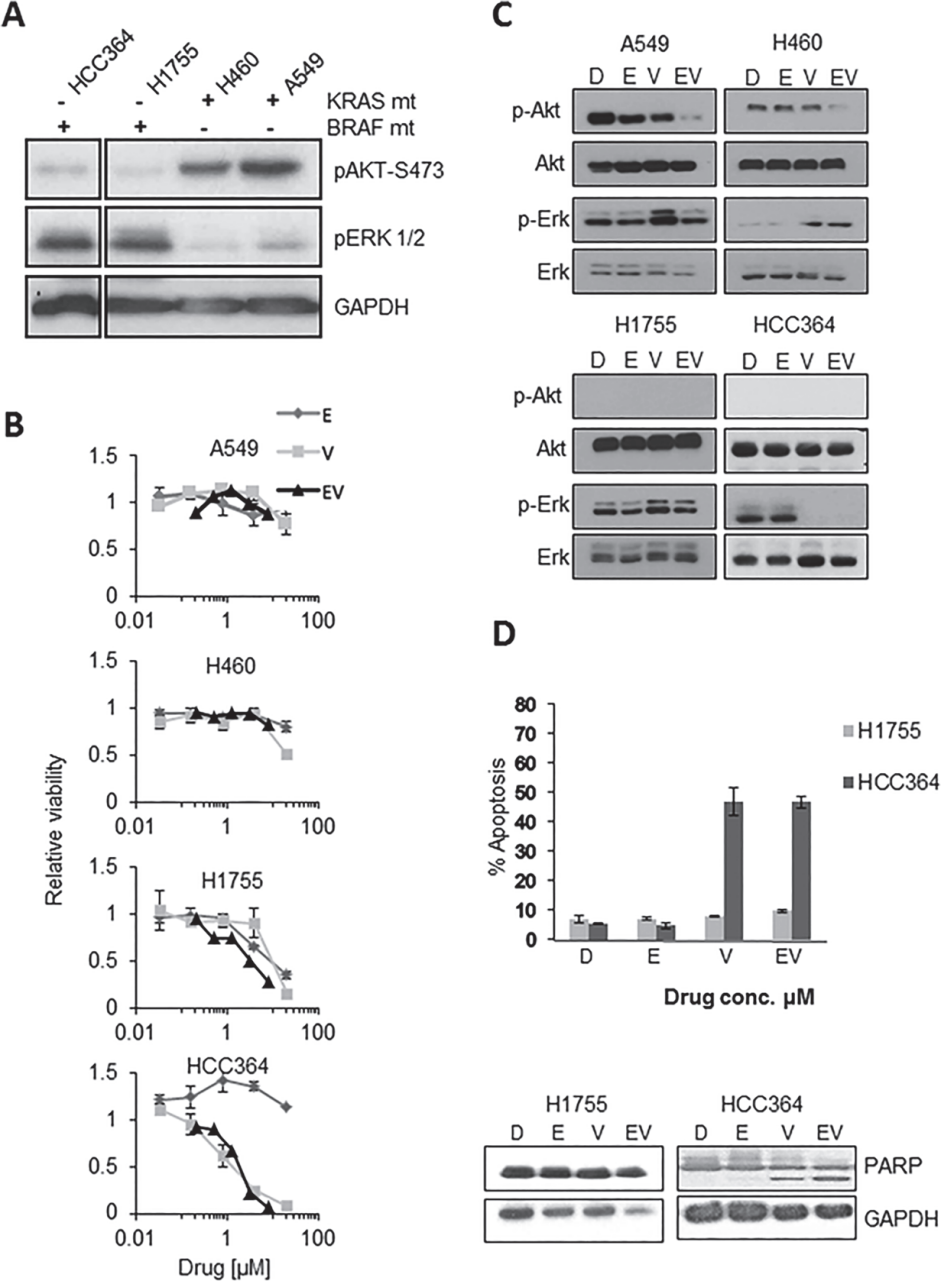
Investigating the effects of the vemurafenib and erlotinib combination in BRAF and KRAS mutant NSCLC cells. **A**: Western blot of phosphorylated AKT and ERK signaling in native untreated cell lines ([+] mutated; [–] wildtype). All lanes are from the same gel and break was created to include only relevant cell lines **B:** A549, H460, H1755 and HCC364 cell lines treated with D (DMSO) or indicated drugs, E (erlotinib), V (vemurafenib), and EV (erlotinib-vemurafenib) were analyzed after 72h by a MTS assay. The IC50 for Vemurafenib in HCC364 was 0.8 μM. **C:** Western blot of different NSCLC cell lines, showing changes in p-ERK, p-AKT and PARP with vehicle D (DMSO), E (erlotinib) 1.6 μM, V (vemurafenib) 1.6 μM, EV (erlotinib+vemurafenib) 1.6/1.6, μM after 24h treatment. **D:** Apoptosis by flow cytometry 48h post treatment with D (DMSO), E (erlotinib) 1.6 μM, V (vemurafenib) 1.6 μM, EV (erlotinib/vemurafenib 1.6/1.6 μM). Western blot, post 48h, supporting PARP cleavage with V and EV in HCC364 but not H1755 cells.

The deregulation of both RAF/MAPK and AKT/MTOR signaling pathways is thought to play a critical role in carcinogenesis and tumor progression in NSCLC [22]. Numerous components of these signaling pathways can act as molecular targets in cancer therapeutics, but it is essential to know the presence of these aberrations in cancer cells for clinical implication [23]. We first determined the baseline expression of phosphorylated (p)-AKT and p-ERK 1/2 in our cell lines (Fig. 1A). Phosphorylated ERK signal was detected in all 4-cell lines used in our experiments, suggesting that the ERK pathway plays an important role in cancer development in KRAS or BRAF mutated cells. Drugs that target this pathway may prove efficacious. However, p-AKT was faintly detected in BRAF mutated cells, HCC364 and H1755 when compared to A549 and H460 cell lines, as shown in Fig. 1A. This may suggest that the AKT signaling pathway may not play as crucial a role in BRAF mutated cells.

### Erlotinib and vemurafenib

**Efficacy of combination therapy with erlotinib and vemurafenib.** The combination of an EGFR targeted monoclonal antibody (cetuximab) and BRAF (vemurafenib) inhibition had shown efficacy in colon cancer cells with BRAF mutation [15]. We sought to determine if a similar concept of combining BRAF and EGFR inhibitions could be effective in NSCLC cell lines. A549, H460, H1755 and HCC364 cells were treated with varying concentrations of erlotinib, vemurafenib, or the combination of erlotinib and vemurafenib (5-fold serial dilutions for single agents and 2.5 fold dilutions of 1:1 ratio of the combination). Cell proliferation assays showed that only HCC364 cells were sensitive to vemurafenib and the combination of vemurafenib and erlotinib was not effective (Fig. 1B). H1755 cells were less sensitive to vemurafenib and no significant synergy was observed with the combination of erlotinib and vemurafenib. Vemurafenib was ineffective in KRAS mutated cells (A549, H460). Additionally, the lack of synergy between erlotinib and vemurafenib was confirmed using the BLISS method (S2 Fig.) [24].

The oncogenic signaling changes after treatment for 24 hours with the combination of vemurafenib plus erlotinib (1.6 μM/1.6 μM) were analyzed by immunoblot assay (Fig. 1C). Vemurafenib decreased phosphorylation of ERK in HCC364 cells only without a subsequent increase in activated AKT. This finding suggested that the lack of synergy between erlotinib and vemurafenib seen in proliferation assays is due to the ERK-AKT relationship seen in colon cancer and is not present in the HCC364 cells. This is probably not related to the choice of EGFR inhibitor (cetuximab vs. erlotinib). Vemurafenib alone induced activation of ERK signaling in non-BRAF mutated cells, A549 and H460. No significant change in activated ERK was noted in H1755 cells following vemurafenib treatment. Interestingly, we observed that the combination of vemurafenib and erlotinib resulted in a decrease in activated AKT in KRAS mutated cells (Fig. 1C).

Finally, we assessed changes in apoptosis after erlotinib and vemurafenib treatments alone or in combination. HCC364 cells were sensitive to vemurafenib-induced apoptosis and PARP cleavage, while H1755 cells were resistant (Fig. 1D). The mechanism of apoptosis was assessed by immunoblot assays after 48 hours of treatment with vemurafenib and it caused an increase in BIM (pro-apoptotic), when compared to DMSO. A decrease in MCL-1 (anti-apoptotic) was also seen with vemurafenib but no change was seen in BCL-xL (anti-apoptotic), BCL-2 (anti-apoptotic), BAK (pro-apoptotic), and BAX (pro-apoptotic) when compared to DMSO (S3 Fig.).

**Fig 2:**
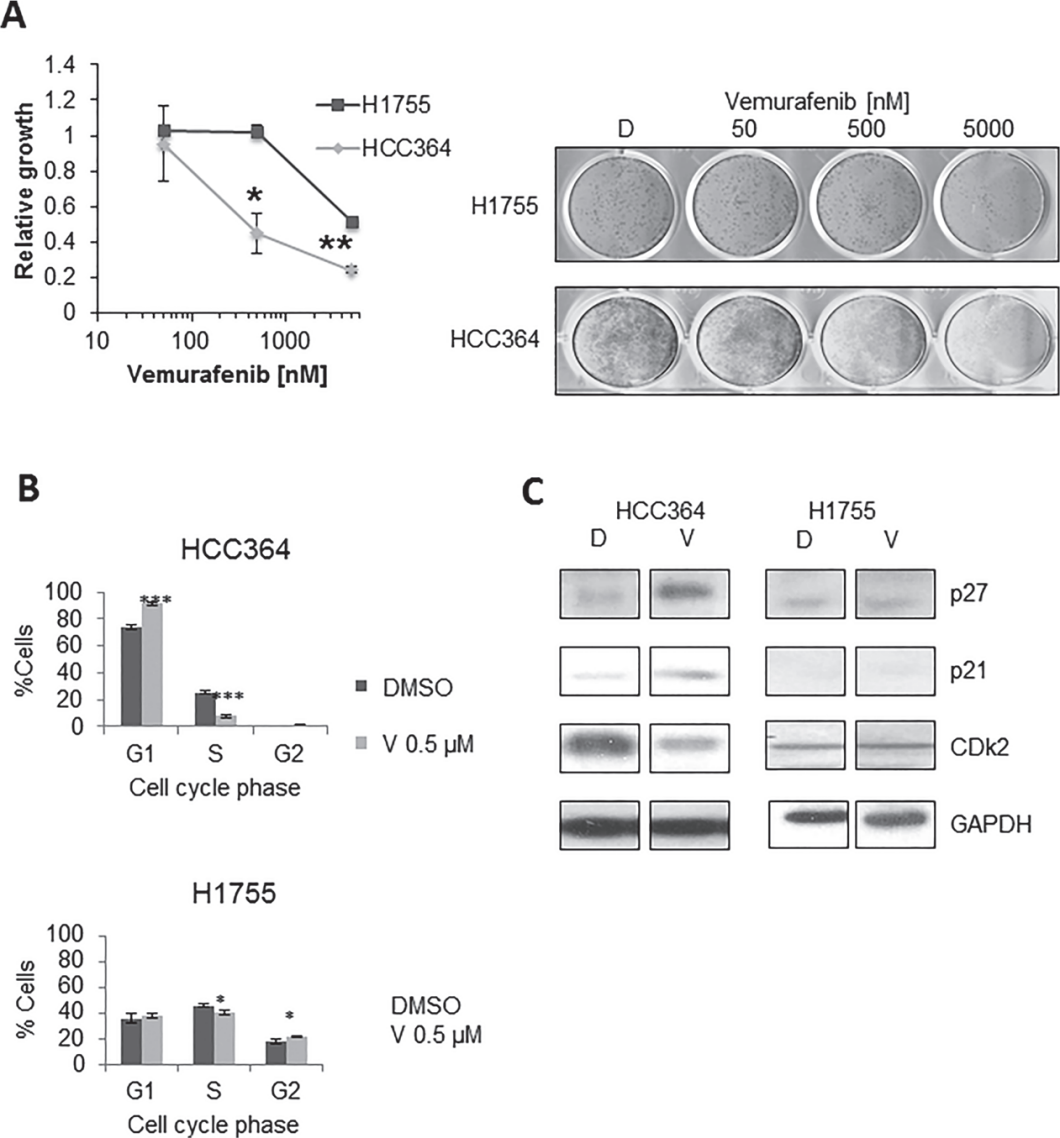
Effects of single-agent vemurafenib on BRAF mutated NSCLC cells. **A:** Long-term growth assay, 7 days post treatment with vehicle-D (DMSO), vemurafenib 50 nM, 500 nM, 5000 nM in both HCC364 and H1755 cells. HCC364 cells were more sensitive to vemurafenib. * p< 0.05; *** p<0.001 when compared to DMSO. **B:** Cell cycle analyses by flow cytometry 24h post treatment with DMSO, V (vemurafenib) in HCC364 and H1755 cells. Cell cycle phases shown are G1, S and G2 phase. V induces cell cycle arrest in HCC364 cells, as evidenced by an increase in G1 and a significant decrease in S phase. **C:** Western blot at 48 hours post treatment supporting the evidence for G1 arrest, showing increase in p27 and decrease in CDk2 with V (vemurafenib) when compared to D (DMSO). No effect was seen in H1755 cells. All lanes for HCC364 and all lanes for H1755 are from the same gel. The break has been created to remove erlotinib treated lanes.

**Effect of single agent vemurafenib in BRAF mutated NSCLC cell lines.** Using a long-term growth assay vemurafenib was found to be effective in BRAF-V600E mutated HCC364 cells and not in non-V600E BRAF mutated H1755 cells (Fig. 2A). We then wanted to further evaluate the molecular mechanism behind this anti-tumor activity. We analyzed changes in the cell cycle 24 hours after treatment with vemurafenib in both HCC364 and H1755 cells. In HCC364 cells, vemurafenib caused a significant increase in the number of G1 cells with a subsequent decrease in S phase cells, supporting G1 arrest in V600E mutated cells. Vemurafenib, however, was ineffective at causing a G1 block in H1755 cells (Fig. 2B). Immunoblotting showed changes in cell cycle proteins in HCC364 cells, after vemurafenib treatment including, downregulation of CDK2 expression and upregulation of p21 and p27 expressions (Fig. 2C).

## Trametinib and vemurafenib

**Fig 3:**
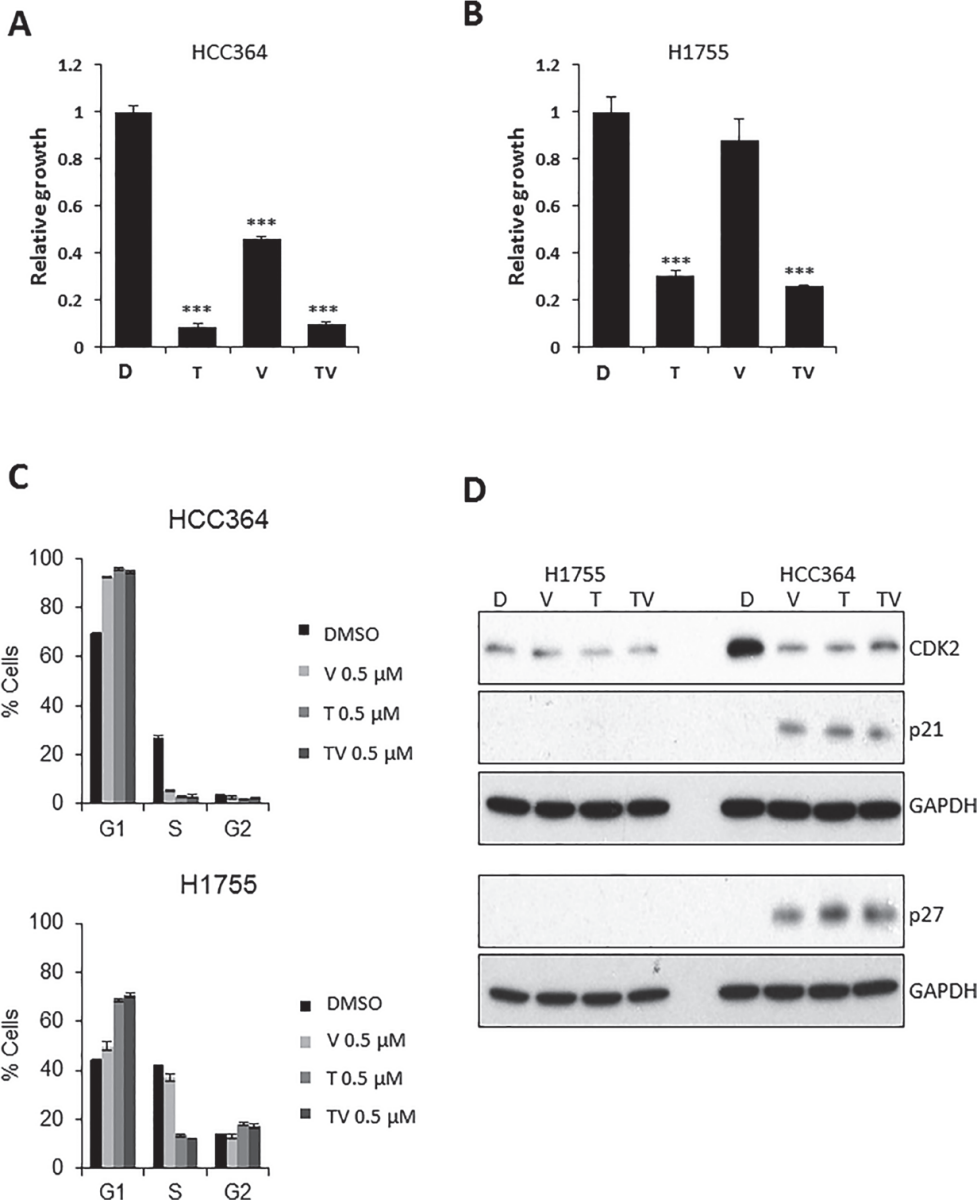
Growth and cell cycle effects of trametinib and vemurafenib combination on BRAF mutated NSCLC cell lines. **A, B:** Long-term growth assay, 7 days post treatment with vehicle-DMSO (D), V (vemurafenib) 1 μM, T (trametinib) 1 μM and TV (trametinib + vemurafenib, 1 μM each) in HCC364 **(A),** and H1755 cells **(B). C:** Cell cycle analyses by flow cytometry in HCC364 and H1755 cells after 24 hours treatment with D, V 0.5 μM, T 0.5 μM, TV 0.5/0.5 μM. **D:** Western blot after 24h of H1755 and HCC364 treated like in C. (***p<0.001 when compared to DMSO).

**Effect of combination treatment with trametinib and vemurafenib.** Treatment with combination of MEK inhibitor and BRAF inhibitor has been effective in advanced melanoma with BRAF-V600 mutation but the effect of MEK inhibitor or this similar combination has not been explored in BRAF mutated NSCLC [12]. We analyzed if the addition of trametinib to vemurafenib would show any efficacy in BRAF mutated cells, H1755 and HCC364. Trametinib was more effective at inhibiting growth after seven days in both cell lines compared to vemurafenib, however the combination of the two drugs was not significantly different than trametinib alone (Fig. 3A,B). We evaluated differences in cell cycle for both BRAF mutant NSCLC cells after a 24-hour treatment with DMSO, vemurafenib, trametinib and the combination (Fig. 3C). Both vemurafenib and trametinib were effective in causing G1 arrest as evidenced by a significant increase in G1 phase and a decrease in S phase cells compared to DMSO treatment. However, the combination was not more effective than either of the single agents alone. The cell cycle arrest observed with flow cytometry in the HCC364 cells was confirmed by immunoblot assay for cell cycle proteins after 24 hours of treatment with respective agents. It showed downregulation of CDK2 with upregulation of p21 and p27 with vemurfenib, trametinib and the combination when compared to the DMSO (Fig. 3D). However there was no difference noted in the cells treated with trametinib, vemurafenib, or the combination of trametinib plus vemurafenib in the expressions of CDK2, p21 or p27 in these cells (Fig. 3D). In addition, we did not observe any difference in pJAK2 and pSTAT3 expressions (S4 Fig.) Interestingly, H1755 cells did not exhibit a similar trend in CDK2 or p21 and p27 in spite of G1 arrest and decrease in S phase seen with flow cytometry (Fig. 3D). This makes us believe that there is another mechanism involved in G1 arrest, which is independent of CDK2 downregulation in these cells.

**Fig 4:**
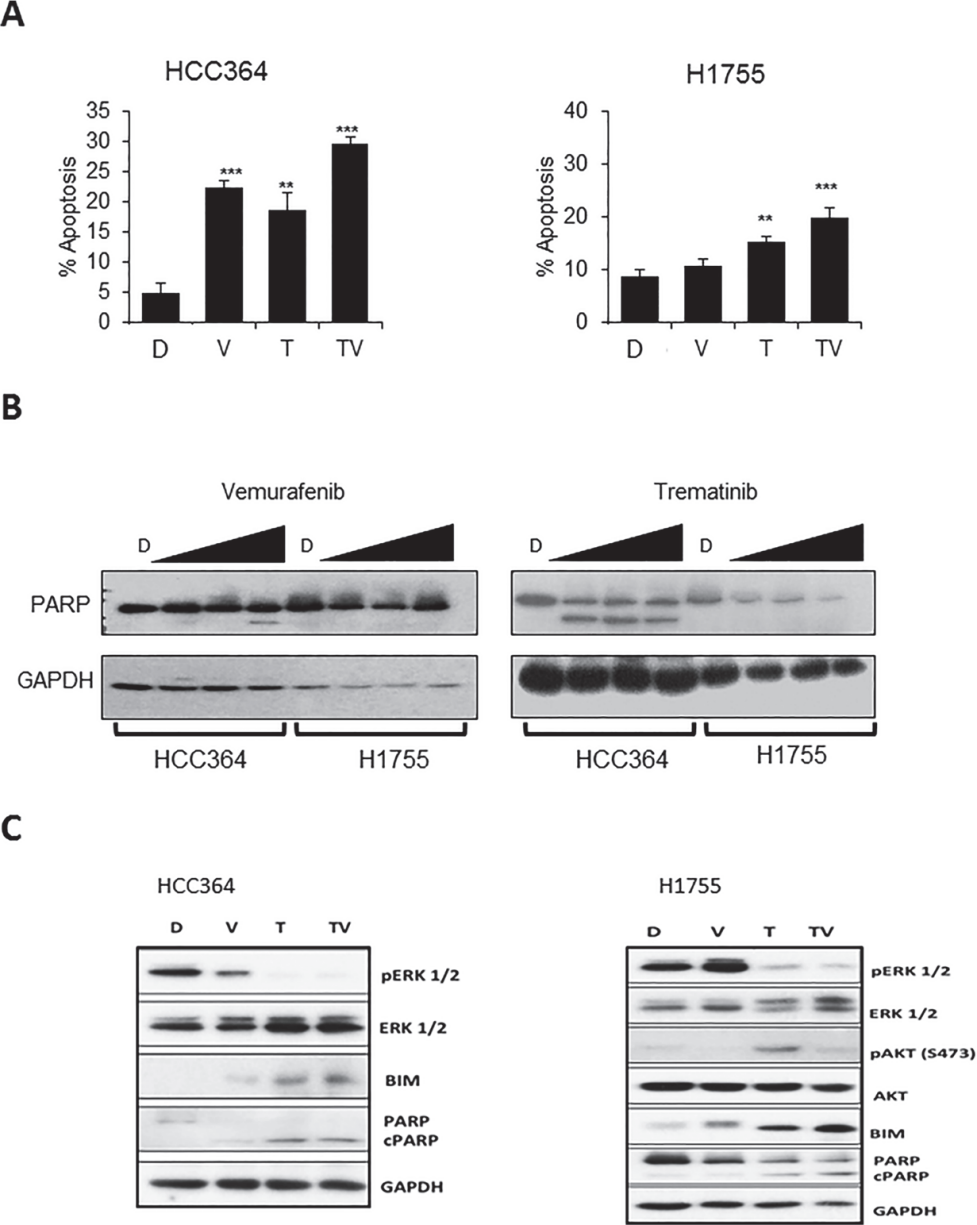
Anti-apoptotic effects of trametinib and vemurafenib in BRAF mutated NSCLC cell lines. **A:** Apoptosis by flow cytometry, 48h post treatment with vehicle-D (DMSO), V (vemurafenib 1 μM), T (trametinib 1 μM), TV (trametinib + vemurafenib 1/1 μM) in HCC364 and H1755 cells. (*p<0.05;** p <0.01; *** p<0.001). **B:** Western blot of cleaved PARP in HCC364 and H1755, 48h post treatment with D (DMSO) and varying doses of vemurafenib (0.25, 0.5, 1μM) and trametinib (0.25, 0.5, 1μM) **C:** Western blot showing changes in ERK, AKT, BIM, PARP cleavage, 48h post treatment with vehicle D (DMSO), V (vemurafenib 1 μM), T (trametinib 1 μM), TV (trametinib + vemurafenib 1/1μM) in HCC364 and H1755 cells. Four lanes were treated in duplicates for each cell lines and only the left 4 lanes of each cell lines have been shown in the figure.

We further explored the apoptotic effect of the vemurafenib and trametinib combination in BRAF mutated NSCLC cells. Trametinib induced apoptosis in both BRAF mutated cells lines. The combination of vemurafenib and trametinib caused significantly higher apoptosis than either single agent in HCC364 and H1755 (Fig. 4A). Apoptosis following various doses of trametinib treatment was confirmed by presence of PARP cleavage in HCC364 cells, whereas a decrease in PARP was seen in H1755 cells without the presence of cleaved PARP (Fig. 4B). The occurrence of apoptosis was further supported by upregulation of pro-apoptotic protein, BIM, in both HCC364 and H1755 cells upon treatment with trametinib. The combination caused greater increase in BIM when compared to trametinib alone. This observation suggests that the combination of vemurafenib and trametinib is better at promoting apoptosis than trametinib alone in BRAF mutated cells (Fig. 4C). No significant changes were observed in expression of BCL-2, BCL-xl, MCL-1, BAX and BAK.

**Trametinib as a single agent in BRAF mutated NSCLC.** The MEK inhibitor, selumetanib has been shown to downregulate ERK signaling in preclinical lung models with KRAS mutated NSCLC, but a similar effect has not been demonstrated in BRAF mutated NSCLC [25]. We wanted to compare the oncogenic signal alterations resulting from introduction of a BRAF inhibitor, a MEK inhibitor, or the combination of both in BRAF mutated NSCLC. The changes in oncogenic signals were evaluated by performing an immunoblot assay to assess changes in apoptosis signals in both cells with vemurafenib vs. trametinib vs. the combination at 48 hours treatment with 1 μM dose of single agents and 1:1 ratio of the combination, 1:1μM doses each. We observed that treatment with trametinib caused a significant suppression in phosphorylation of ERK when compared to DMSO or vemurafenib alone in both HCC364 and H1755 cells (Fig. 4C). The combination of trametinib and vemurafenib was not better than trametinib alone. In addition we noticed that pAKT was not elevated in HCC364 cells with the use of either single agents or combination (p-AKT was faintly detected in HCC364 cells and the expression did not change-the immunoblot is not shown). However, we saw an increase in p-AKT expression with the use of single agent trametinib in non-V600E BRAF mutated H1755 cells and interestingly the combination of trametinib and vemurafenib did not show an increase in p-AKT when compared to DMSO, suggesting that perhaps p-AKT pathway may play a role in resistance to treatment with single agent trametinib. Trametinib is also effective in causing apoptosis in both HCC364 and H1755 cells (Fig. 4A). In addition, trametinib also caused G1 arrest in both BRAF mutated cells (Fig. 3C). Although the changes in cell cycle proteins, downregulation of CDK2 and upregulation of p21 and p27 expressions, were only seen in HCC364 cells.

## Discussion

BRAF pathway plays an important role in tumorigenesis in NSCLC, BRAF inhibitors, vemurafenib and dabrafenib have shown only modest efficacy in BRAF-V600E mutant cancers [11,13]. Despite targeting this specific mutation, the use of dabrafenib, in NSCLC with BRAF-V600E mutation has resulted in only a 40% response rate along with a disappointing 30% disease progression [11]. These results reflect that BRAF inhibition alone is not an ideal treatment option and newer strategies for optimized and effective treatment of this select group of patients are warranted. This hypothesis is also supported by a clinical study in melanoma showing a significant advantage in progression free survival with combination of dabrafenib plus trametinib vs. dabrafenib alone [12]. Our in-vitro experiments did confirm that vemurafenib is effective in inhibiting growth, decreasing ERK signaling, and inducing apoptosis in the BRAF V600E mutant cell line HCC364, while in non-V600E BRAF mutant cell line, H1755, was not as sensitive. Prahallad *et al.*, have demonstrated increased activity by combined treatment of the EGFR inhibitor cetuximab with vemurafenib by linking ERK and AKT signaling through the intermediary CDC25C in BRAF mutated colon cancer cells [15]. However, our results reflect that the combination of erlotinib and vemurafenib is not effective in NSCLC cells. Although we were unable to detect CDC25C in the BRAF mutant cell lines HCC364 (V600E) and H1755 (non-V600E), these cells did not respond to ERK inhibition with enhanced AKT activation suggesting that ERK and AKT signaling are not linked through CDC25C in these cells.

Vemurafenib was able to modulate apoptosis related proteins and the cell cycle [26,27]. We found that treatment with vemurafenib resulted in decreased levels of the anti-apoptotic protein MCL-1 and elevated levels of the pro-apoptotic protein BIM in NSCLC cell lines. MCL-1 loss along with increased BIM [28–30], leads to apoptosis induction, and increased levels of BIM can also initiate apoptosis [31–33]. Additionally, the cell cycle-related protein, CDK2, was downregulated while cell cycle inhibitory proteins, p21 and p27, were upregulated following vemurafenib treatment in HCC364 cells, resulting in growth arrest. We speculate that these protein expression changes are probably related to the reduced activity of ERK [34,35], since similar effect was seen upon treatment with trametinib (Fig. 3D). This strengthens our hypothesis that modulation of BIM and MCL-1 are related to the reduced activity of ERK in HCC364 cells. Unfortunately, H1755, BRAF-non-V600E cells, showed G1 cell cycle arrest dependent upon downregulation of ERK by treatment with trametinib. Cell cycle arrest occurred without alteration of the cell cycle proteins, CDK2, p21 and p27. This suggests that H1755 cells may have a different mechanism involved in G1 arrest independent of CDK2 check point in the cell cycle.

Our results show trametinib is effective in causing apoptosis in both BRAF V600E and non-V600E mutated cells. Trametinib was potent in decreasing ERK signaling in BRAF mutant cells, and this suppression is more pronounced with trametinib when compared to vemurafenib even in BRAF V600E mutated cells. The long-term growth assay results also demonstrate that trametinib has better efficacy as a single agent compared to vemurafenib in BRAF V600E cells but this could be as a result of a shorter half-life of vemurafenib [36,37]. Interestingly, treatment with single agent trametinib caused upregulation of AKT signaling in BRAF non-V600E cells only, suggesting a potential escape mechanism for development of resistance to treatment with MEK inhibitor alone. This may provide an insight to development of resistance to MEK inhibitors. The AKT pathway did not appear to be upregulated when BRAF non-V600E cells were treated with the combination of trametinib and vemurafenib, suggesting that the combination therapy may be superior to each of the two single agents in this group as well. The mechanistic relationship between the ERK inhibition and AKT activation in BRAF mutant NSCLC by MEK inhibitor needs to be further evaluated. We also showed for the first time that the combination treatment caused a significant increase in the upregulation of BIM in both V600E and non-V600E cells, thus playing a significant role in apoptosis [31]. The combination treatment also caused small but a significant augmentation in apoptosis in BRAF mutated cells when compared to single agent, suggesting the rationale for using combination of BRAF and MEK inhibitor in this select group. In addition, the combination of trametinib and vemurafenib could overcome resistance to trametinib alone and thus better than single agent trametinib. Lupin et al. have highlighted the possible mechanism behind the resistance in BRAF-V600E mutated NSCLC cells to the BRAF inhibitor [38]. The two discrete molecular mechanisms for acquiring BRAF inhibitor resistance include loss of full-length in BRAF-V600E coupled with the expression of an aberrant form of BRAF-V600E that retains the RAF pathway dependence and constitutive autocrine EGFR signaling driven by c-Jun–mediated EGFR ligand expression. Our results support the hypothesis of BRAF- mutation independent activation of MAPK pathway leading to the development of resistance to BRAF inhibitor when treating BRAF-V600E mutated NSCLC. In addition, the side-effect and toxicity profile (19% of cutaneous squamous cell carcinoma in BRAF inhibitor arm *vs*. 7% in the combination arm in melanoma patients) favors the combination therapy [12]. Presently, the clinical benefit of combined BRAF and MEK inhibition has not been explored in advanced NSCLC. Our in-vitro data suggests that the combination of trametinib and vemurafenib may be a better therapeutic strategy than either single agent in BRAF mutated NSCLC. It seems that the MEK inhibitor is at least equally effective when compared to BRAF V600E specific inhibitor in BRAF V600E mutated NSCLC and can be an alternative therapeutic option for this select group of patients. Immunoblot results reflect that ERK inhibition is better with the MEK inhibitor as compared to the BRAF V600E inhibitor and it may prove to be more efficacious when treating patients with BRAF mutated tumors. Our encouraging data forms the rationale for the clinical evaluation of trametinib *vs*. the combination of trametinib plus vemurafenib for patients with advanced BRAF mutated NSCLC patients.

## Supporting Information

S1 Fig Conformation of BRAF V600E and G469A mutations in HCC364 and H1755 cells, respectively. DNA extracted from HCC364 and H1755 cells was analyzed for BRAF mutations using the SNaPshot fragment analysis method. Cell lines were analyzed with a water only sample (-C), normal human DNA (Wt, Promega, Madison Wisconsin) and Wt DNA with positive control primers (+C). Wt panels show wildtype alleles and arrows indicate mutant alleles in cell lines panels. HCC364 and H1755 cells were confirmed to be heterozygous for their respective mutations.
(TIFF)

S2 Fig Investigating the synergy with erlotinib and vemurafenib in BRAF mutated NSCLC cell lines. Bliss sum in both HCC364 and H1755 cells: Synergy using a 5x5 matrix was evaluated using a CellTitre-Glo assay and analyzed using Bliss additive model. Positive value suggests synergy, and more positive the bliss sum is more synergy is seen. HCC364 cells, some higher doses of erlotinib (ERL) when combined with vemurafenib (Vem) hinted synergy but this was not observed consistently with different doses, suggesting there no effective synergy with this combination.
(TIFF)

S3 Fig Western blot, showing signal changes in the apoptosis related proteins in HCC364 and H1755 cells post 48h of treatment with DMSO, E (erlotinib), V (vemurafenib), EV (erlotinib plus vemurafenib). The drug concentration was chosen based upon the CellTiter-Glo. No significant changes in BCL-xL (anti-apoptotic), BCL-2 (anti-apoptotic), BAK (pro-apoptotic), and BAX (pro-apoptotic).
(TIFF)

S4 Fig Western blot, showing changes in pJAK2 and pSTAT3 in BRAF mutated NSCLC cell lines. It shows no signal changes in p-JAK2 and pSTAT3 in H1755 and HCC364 cells post 2h of treatment with D (DMSO), V (vemurafenib 0.5 μM), T (trametinib 0.5 μM), TV (trametinib plus vemurafenib 0.5/0.5 μM).
(TIFF)
