# Supplementary figures and images for: Trametinib with or without Vemurafenib in BRAF Mutated Non-Small Cell Lung Cancer
Source: PLoS One. 2015 Feb 23;10(2):e0118210. doi: 10.1371/journal.pone.0118210 (PMC4338247; doi:10.1371/journal.pone.0118210)

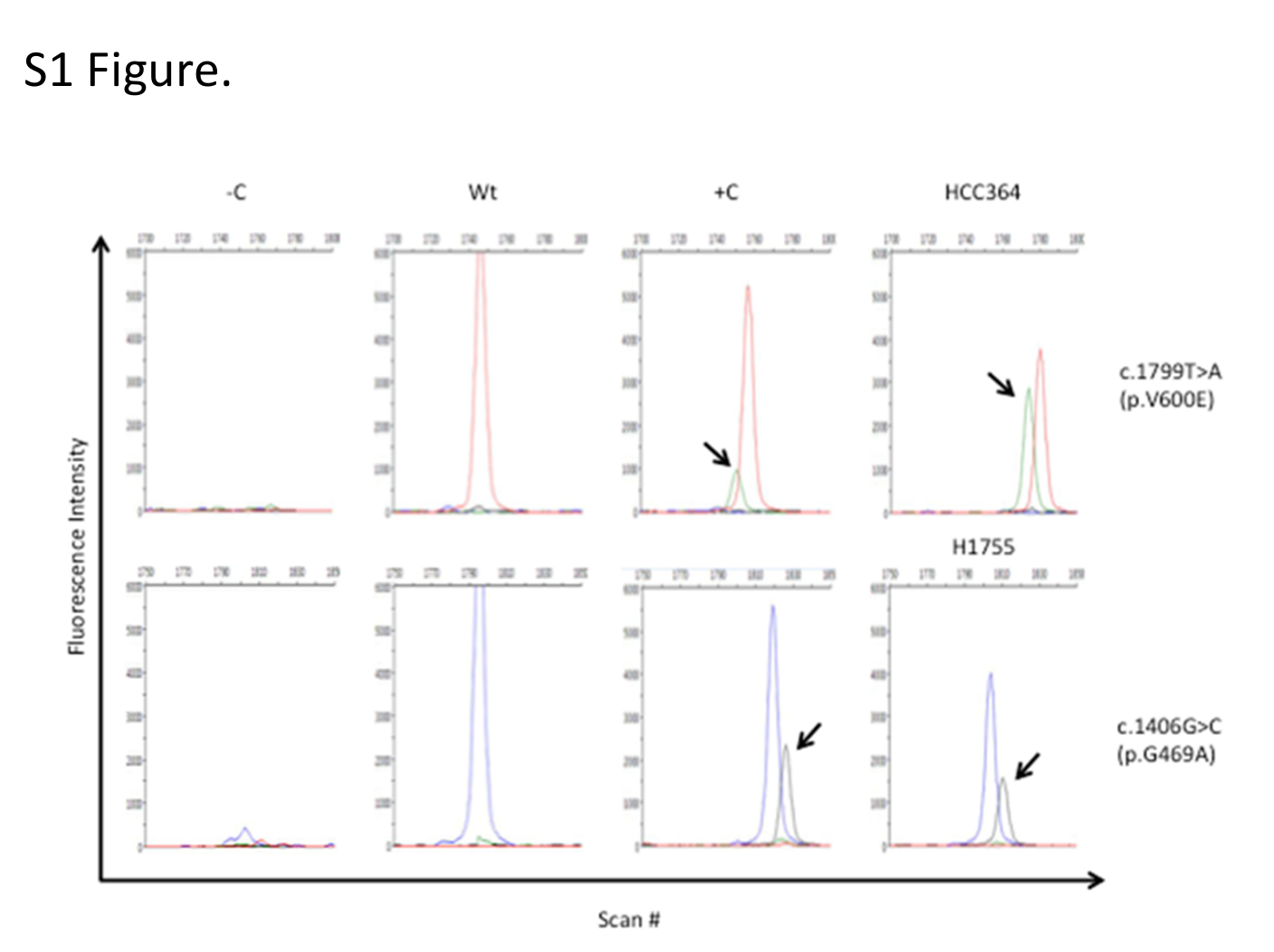

Supplement: S1 Fig — DNA extracted from HCC364 and H1755 cells was analyzed for BRAF mutations using the SNaPshot fragment analysis method. Cell lines were analyzed with a water only sample (-C), normal human DNA (Wt, Promega, Madison Wisconsin) and Wt DNA with positive control primers (+C). Wt panels show wildtype alleles and arrows indicate mutant alleles in cell lines panels. HCC364 and H1755 cells were confirmed to be heterozygous for their respective mutations. (TIFF) [file pone.0118210.s001.tiff]

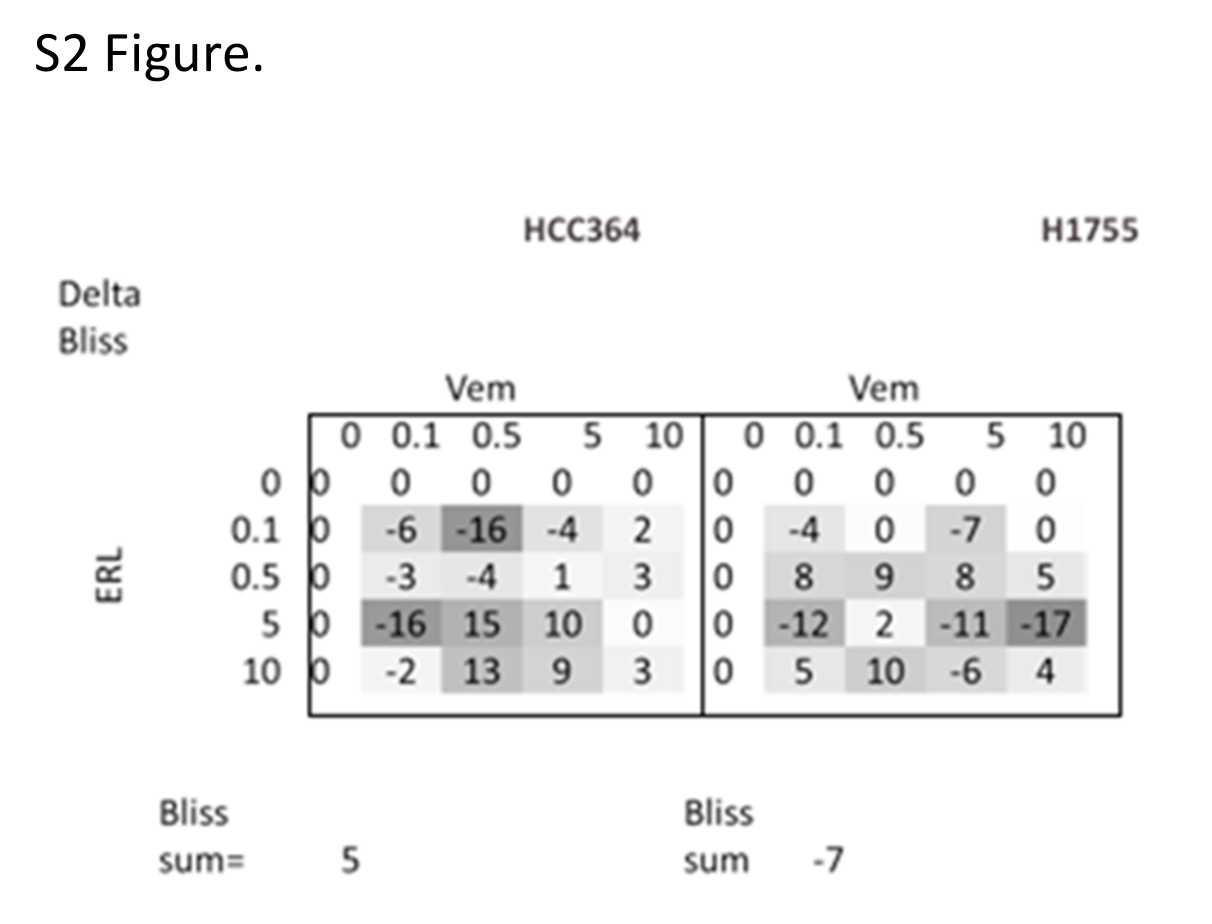

Supplement: S2 Fig — Bliss sum in both HCC364 and H1755 cells: Synergy using a 5x5 matrix was evaluated using a CellTitre-Glo assay and analyzed using Bliss additive model. Positive value suggests synergy, and more positive the bliss sum is more synergy is seen. HCC364 cells, some higher doses of erlotinib (ERL) when combined with vemurafenib (Vem) hinted synergy but this was not observed consistently with different doses, suggesting there no effective synergy with this combination. (TIFF) [file pone.0118210.s002.tiff]

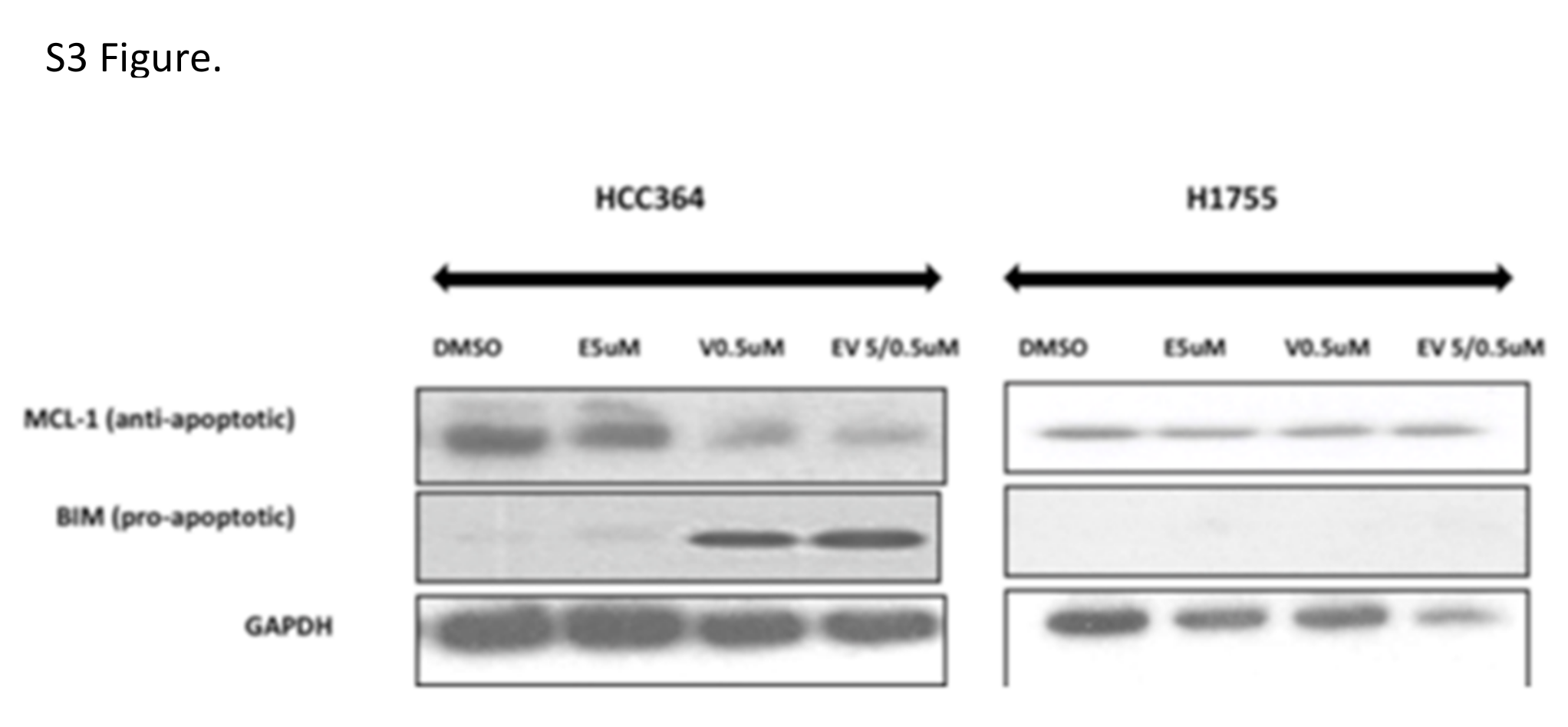

Supplement: S3 Fig — The drug concentration was chosen based upon the CellTiter-Glo. No significant changes in BCL-xL (anti-apoptotic), BCL-2 (anti-apoptotic), BAK (pro-apoptotic), and BAX (pro-apoptotic). (TIFF) [file pone.0118210.s003.tiff]

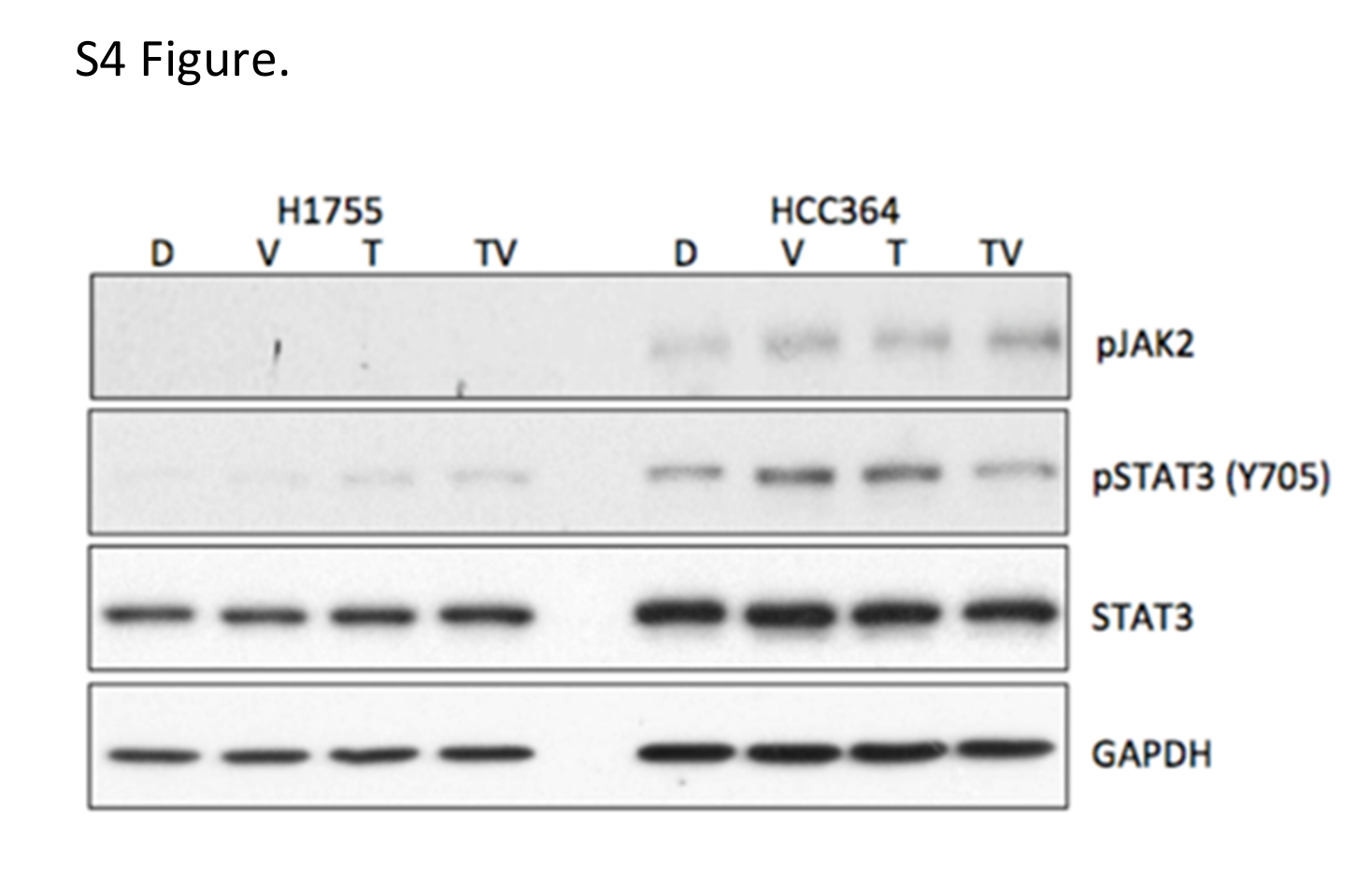

Supplement: S4 Fig — It shows no signal changes in p-JAK2 and pSTAT3 in H1755 and HCC364 cells post 2h of treatment with D (DMSO), V (vemurafenib 0.5 μM), T (trametinib 0.5 μM), TV (trametinib plus vemurafenib 0.5/0.5 μM). (TIFF) [file pone.0118210.s004.tiff]
